# Bennet on the Uterus

**Published:** 1854-02

**Authors:** 


*Bennet on the Uterus.* Philadelphia: Blanchard & Lea.

THIS work has been in the hands of the profession since 1845, and both in England and this country has given rise to a lively discussion.

We are not among those who advocate an indiscriminate use of the speculum, in many, in most instances we believe that a diagnosis may be made without subjecting the patient to such a sacrifice, and yet we think that in the hands of a judicious and intelligent physician the instrument may sometimes be of great value. Like everything else, however, it must be made a hobby and rode to death before its real value will be determined.

Dr. Bennet's work is elaborate and contains perhaps the most clear and definite descriptions of uterine disease with which we are acquainted.

For sale by Keen & Bro's. J.

